# An autonomous wheelchair with health monitoring system based on Internet of Thing

**DOI:** 10.1038/s41598-024-56357-y

**Published:** 2024-03-11

**Authors:** Lei Hou, Jawwad Latif, Pouyan Mehryar, Stephen Withers, Angelos Plastropoulos, Linlin Shen, Zulfiqur Ali

**Affiliations:** 1 Healthcare Innovation Centre, School of Health & Life Sciences, Teesside University, Middlesbrough, TS1 BX UK; 2 Zhejiang Lab, Research Center for Frontier Fundamental Studies, Hangzhou, 311121 China; 3 Innovative Technology and Science Ltd, Hildersham Road, Cambridge, CB21 6DR UK; 4 College of Computer Science and Software Engineering, Shenzhen University, Shenzhen, 518060 China

**Keywords:** Autonomous driving, Healthcare monitoring, Internet of Things, Smart wheelchair, Health care, Engineering

## Abstract

Assistive powered wheelchairs will bring patients and elderly the ability of remain mobile without the direct intervention from caregivers. Vital signs from users can be collected and analyzed remotely to allow better disease prevention and proactive management of health and chronic conditions. This research proposes an autonomous wheelchair prototype system integrated with biophysical sensors based on Internet of Thing (IoT). A powered wheelchair system was developed with three biophysical sensors to collect, transmit and analysis users’ four vital signs to provide real-time feedback to users and clinicians. A user interface software embedded with the cloud artificial intelligence (AI) algorithms was developed for the data visualization and analysis. An improved data compression algorithm Minimalist, Adaptive and Streaming R-bit (O-MAS-R) was proposed to achieve a higher compression ratio with minimum 7.1%, maximum 45.25% compared with MAS algorithm during the data transmission. At the same time, the prototype wheelchair, accompanied with a smart-chair app, assimilates data from the onboard sensors and characteristics features within the surroundings in real-time to achieve the functions including obstruct laser scanning, autonomous localization, and point-to-point route planning and moving within a predefined area. In conclusion, the wheelchair prototype uses AI algorithms and navigation technology to help patients and elderly maintain their independent mobility and monitor their healthcare information in real-time.

## Introduction

An electric-powered wheelchair (EPW) is an assistive technology solution for people with motor disabilities, which gives them independent mobility. An estimated 65 million people worldwide need a wheelchair^1^, and the number of people who are in need of a wheelchair is estimated to increase over 22% in the next decade^2^. There is a high level of demand for wheelchair services for the elderly that is difficult to meet.

The research on EPW started around the 1980s. The prototype wheelchair allowed a person to maneuver within an office building^3^. Since then, many EPWs have been developed and commercialized, such as TinMan^4^, NavChair^5^, Maid^6^, and SPAM^7^ to provide users indoor mobility. However, the traditional type of EPW was controlled by a joystick and was difficult to maneuver by patients with complicated disabilities and mobility impairment due to cerebral palsy, cognitive impairment, and fatigue^8^. For example, patients with Parkinson’s disease often lack the cognitive and physical skills to maneuver the EPW due to perceptual impairments. A study of 65 clinicians reported that between 10 and 40% of their patients could not be equipped with EPW due to sensory disabilities, impaired mobility, or cognitive deficits. These impairments made it difficult to operate a wheelchair safely with the current control functions^9^. Consequently, those individuals who cannot maneuver an EPW independently and safely must be seated in a manual wheelchair and pushed by a caregiver. To solve these problems, academics improved the design of the EPW in three main areas: the assistive technology mechanics, physical interface, and power shared control between the user and the wheelchair^10,11^.

Currently, most autonomous wheelchairs are modified by existing commercially available EPW, with additional facilities to improve maneuverability, locomotion, localization, navigation, and control interface^12^. The smart autonomous wheelchairs have been trialled in hospitals and airports.

In 2016, two prototype autonomous wheelchairs developed by the Singapore-MIT Alliance for Research and Technology Centre were tested in a hospital of Singapore to navigate the hospital’s hallways^13^. The prototype wheelchair created a path map using data from three Lidar sensors. The location of the wheelchair on the map is determined using a localization algorithm. In 2017, an autonomous wheelchair embedded with LIDAR sensors was proposed by Harkishan^14^. This wheelchair can navigate to predefined locations in an unstructured environment. Another model WHILL autonomous wheelchair was developed in 2017 by Panasonic and Whill^13^. This type of wheelchair was premiered at Haneda Airport in Tokyo with further trials in Amsterdam’s Schiphol airport, Abu Dhabi airport and north American airports since 2018^15^. However, these prototypes can only take passengers to predefined locations within the airport or hospital. The maximum luggage carrying capacity of four kilograms cannot fulfill the baggage requirements for most passengers. In addition to autonomous driving, assistive biophysical sensors can be integrated into the wheelchair to check passengers’ vital signs before use.

A robot operating system was used in an autonomous wheelchair for individuals who have difficulty in controlling movements by Grewal^16^. He employed only 2D laser scanners to design a mapping system that enabled the wheelchair to move autonomously. The same approach was used by Wang^17^, but the sensor offered large degree measurements in a narrow space. On the other hand, Surmann utilized a rotatory mechanism and a 2D LiDAR scanner to create a 3D environment map for anti-collision system. Nonetheless, the solution may be insufficient to ensure the safety of the wheelchair user^18^. Furthermore, a wheelchair system developed by Andre can transport inpatients autonomously to their departments by integrating with the hospital information system^19^. However, using this system for private transportation may be challenging, as it requires specific location and path information for departments in the hospital.

Electrically powered wheelchairs should not only provide mobility for advanced stages of disability but also integrate with assistive technology to offer better clinical care. Chronic diseases, such as arthritis, asthma and coronary heart disease, are becoming more prevalent among the elderly and place a high demand for healthcare services^20^. A wheelchair health monitoring system with routine tests can be a cost-effective way for clinicians and caregivers to manage chronic conditions in their patients^21^. The remote monitoring system can improve the management of chronic condition transparency and quality of care for patients while reducing the burden on healthcare facilities, emergency situations, and re-admissions. For example, a biomedical sensing system was integrated into a prototype wheelchair to record users’ pulse rate, respiratory rate and motion states^22^. However, the signal communication and autonomous system were limited by Wi-Fi signals and not viable for outdoor scenarios. Based on that prototype, a home healthcare system for wheelchair users was proposed to connect more sensors in a prototype wheelchair. Similar work was proposed to develop an Intelligent Robotic Wheelchair (iRW)^23^ that integrates telehealth systems to collect vital signs of users in real time. However, there is no effective analysis of these healthcare signals which can be used for remote diagnosis by doctors.

One of the significant limitations for the autonomous telehealth wheelchair is the battery life. The operating of biophysical sensors embedded in the wheelchair is limited by various resources, such as power supply, memory storage and processing capabilities^24,25^. Continuous monitoring sensors produce a large amount of data and consume significant storage memory and transmission power. According to a survey^26^, nearly 80% of the power is consumed during the transmission of data in each sensor node. Therefore, it is essential to develop a lower power design to make the battery last longer. Data compression in sensor nodes before the data transmission provides an adequate method to reduce the size of data. The performance of various data compression algorithms is evaluated based on dataset types.

Lempel–Ziv–Welch (S-LZW) data compression algorithm uses structured data to reduce substantial energy consumption^27^. However, S-LZW is a dictionary-based algorithm that occupies memory for calculation, so it is not suitable for sensors with restricted RAM^25^. Another compression algorithm of Run Length Encoding (RLE) works by removing duplicate data values. Based on RLE, K-RLE was developed to achieve a better compression ratio^28^. Meanwhile, because it concentrates on computing floating-point data, the Minimalist Adaptive and Streaming (MAS) method was recommended as resource efficient^29^. Among them, MAS and S-LZW algorithms have been widely applied in real-time sensing applications, such as monitoring wind speed, rainfall, temperature, humidity, soil moisture, pressure, and battery level^24,30^. The reduction of power consumption during data transmission of the MAS algorithm is between 53.55 and 55.95%, while that of the S-LZW is between 23.41 and 33.97%. To further improve the data compression ratio during transmission, the Minimalist, Adaptive and Streaming R-bit (O-MAS-R) algorithm was proposed.

**Figure 1:**
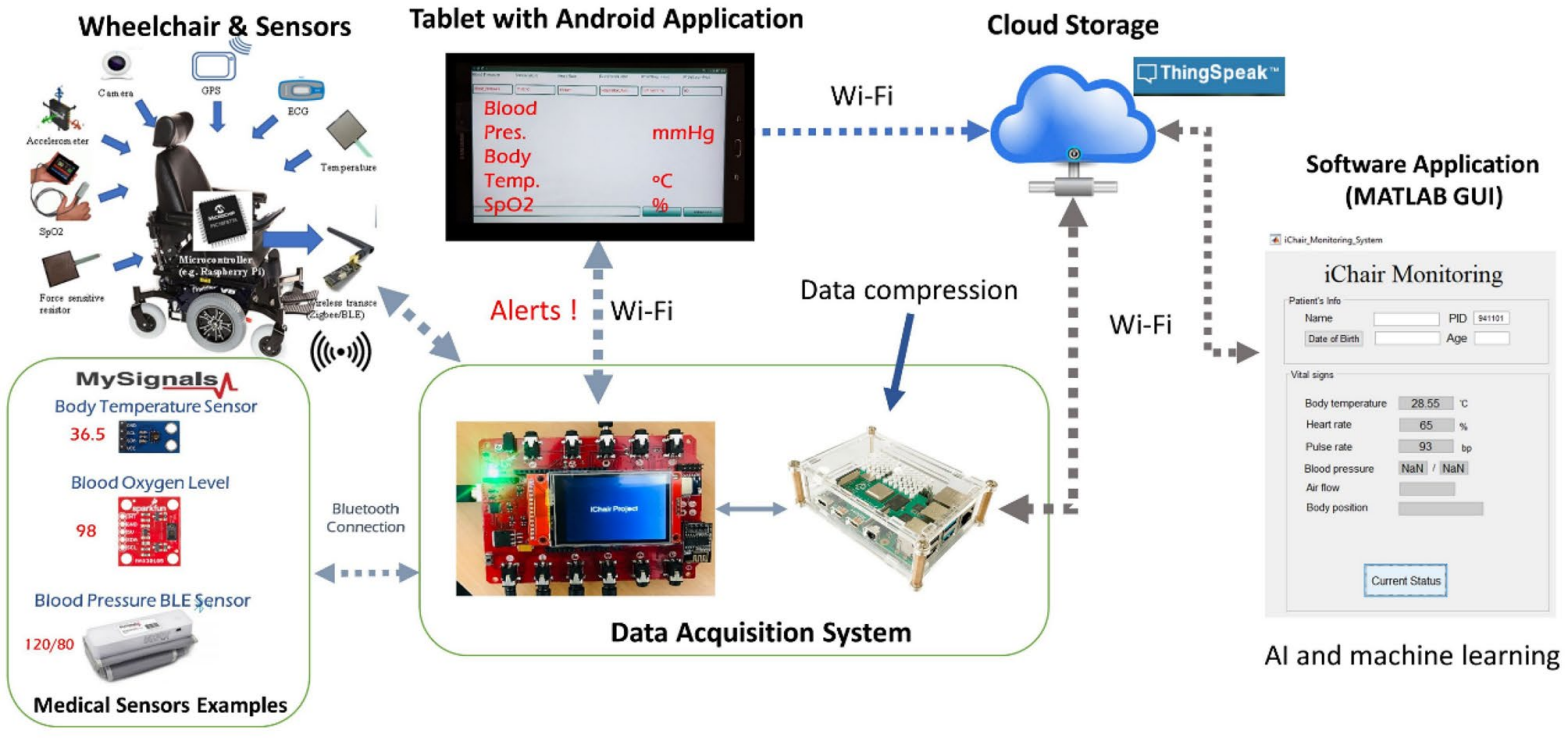
The architecture of the smart wheelchair system. A portable wheelchair is equipped with sensors, cameras, and screens. The data acquisition system processes, compresses and uploads the measurements from biophysical sensors. A MATLAB graphic user interface allows users and doctors access and diagnose the health information in real-time^31^.

In this paper we propose an intelligent autonomous wheelchair (iChair) integrated with telemedicine sensors based on IoT, and the architecture of the wheelchair system is shown in Fig. 1. Various sensors including wireless location, position accelerometer, seat cushion sensors, and biophysical sensors are embedded in the wheelchair to collect users’ physiological and behavioral data in real time. At the same time, an improved data compression algorithm Minimalist, Adaptive and Streaming R-bit (O-MAS-R), is also proposed to achieve a higher compression ratio during the data transmission. To visualize and analyze the data, a user interface was developed to provide telediagnosis, advice and alert to users and caregivers using artificial intelligence algorithms.

## Results

### Wheelchair monitoring interface

**Figure 2:**
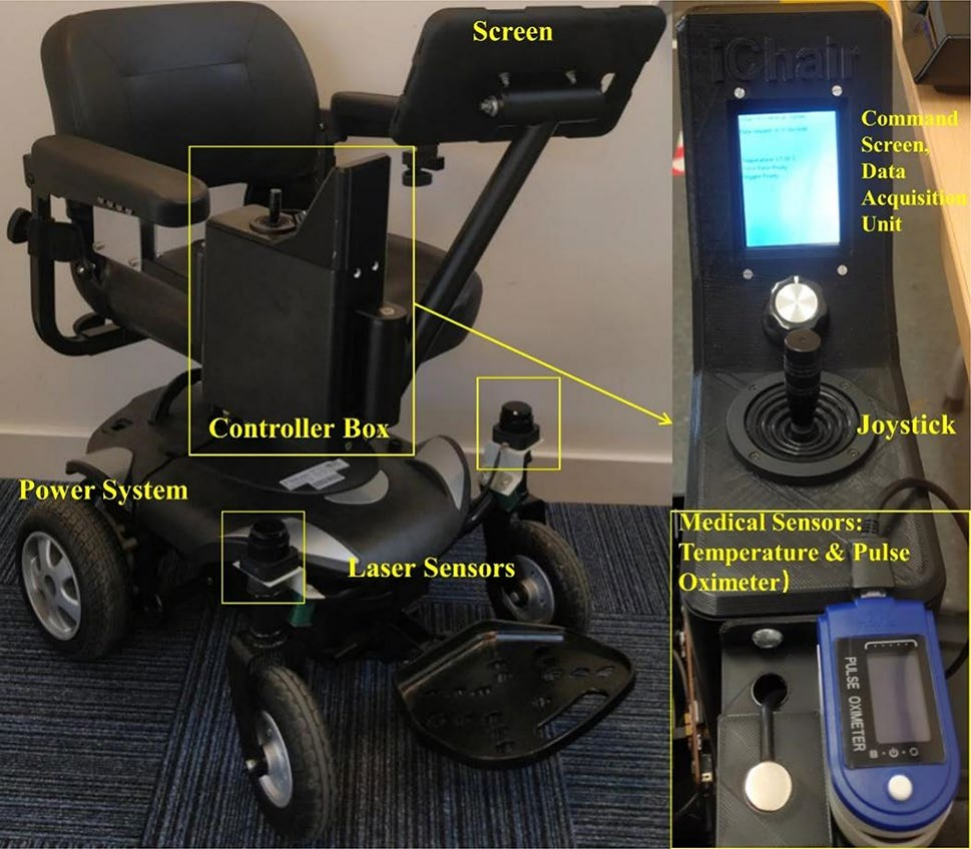
The prototype of the smart wheelchair consists of a controller box, laser sensors, power system, screens, and biophysical sensors^31^.

The handrail of the wheelchair system included three biophysical sensors: pulse oxygen (SpO_2_), blood pressure, and temperature sensors to collect and transmit four kinds of vital signs from users (blood oxygen levels, pulse rate, blood pressure and temperature)^31^, as depicted in Fig. 2.

On the wheelchair as shown in Fig. 2, there are two monitoring interfaces to provide feedback to users: the large screen interface and the handrail screen as depicted in Fig. 2. The screen installed on the handrail of the wheelchair and the remote GUI are for data classification, visualization, and analysis.

The information includes data initialization, measurement, upload status to the cloud, and transmission completion. The duration for each process results in a 40-s cycle, with each set lasting 10 s. The display shows a countdown for each phase, and the timing allows the data from all three biophysical sensors to finish transmitting.

**Figure 3:**
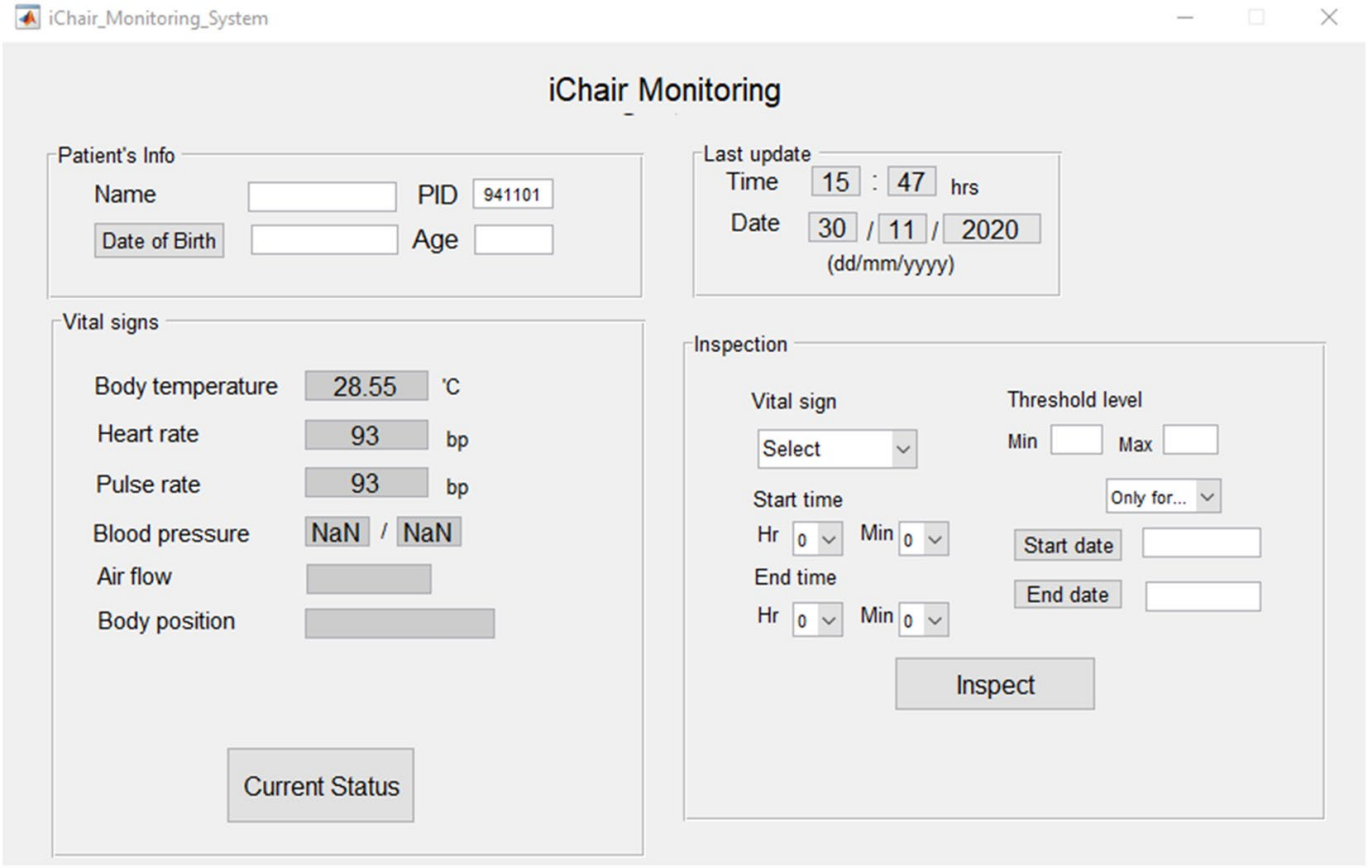
The iChair monitoring interface. The GUI comprises four main sections: patient information, last update, vital signs, and inspection. It allows users and doctors to download and analyze cloud-stored data as well as inspect the data being recorded in real-time.

The GUI, developed in MATLAB and shown in Fig. 3 allows users to download, inspect, and analyze the cloud-stored data once it finishes uploading. In the user interface, access to users’ healthcare data requires a unique Patient Identity number (PID) assigned to each user before experiments. The warning system uses three colors to flag conditions: red, yellow, and blue. The red indicates that the gathered data is above the upper threshold, the yellow shows the data is below the lower threshold, and the blue indicates the measured data is within the thresholds.

Figure 3 shows the iChair monitoring interface comprising four main sections: patient information, last update, vital signs, and inspection. The last update section shows the most recent collecting date and time from the user, and the users’ vital signs appear in the vital signs section. In the inspection section, users can see an aggregated display of their specific vital sign’s information in the past.

### Data compression algorithm

**Figure 4:**
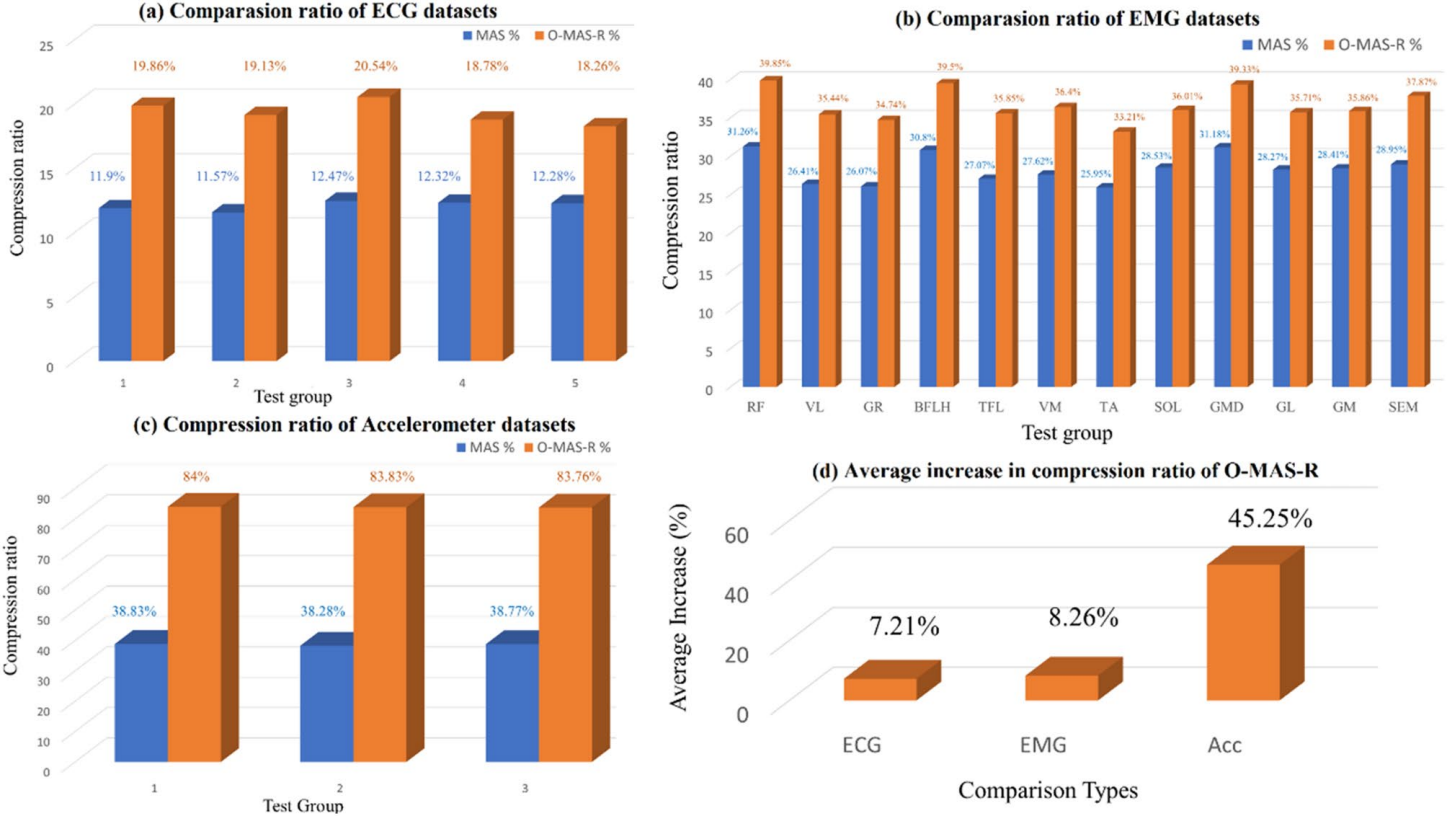
The compression ratio results of MAS and O-MAS-R algorithms are shown in (**a**–**c**), in each figure, x-axis shows the group number, and y-axis is the compression ratio. The average ratio increase of the O-MAS-R algorithm over MAS is shown in (**d**). The compression ratio of (**a**) five ECG datasets, (**b**) twelve EMG datasets and (**c**) three accelerometer datasets are demonstrated.

Both MAS and O-MAS-R compression algorithms were applied to five ECG datasets, twelve EMG datasets, and three accelerometer datasets to evaluate the approaches effectiveness. Figure 4 depicts the compression ratio performance.

In Fig. 4a, the compression results of MAS and O-MAS-R algorithms applied to five ECG datasets are demonstrated. The data in ECG datasets is assigned integer type with two bytes per sample. Each ECG dataset comprises 3,600 samples that occupy 7,200 bytes of memory. Among the simulation results, the group three of O-MAS-R algorithm shows the greatest compression ratio of 20.54%, while the MAS algorithm is 12.47%. For each group, the O-MAS-R method achieves compression ratios of 19.86%, 19.13%, 20.54%, 18.78%, and 18.26% respectively. Meanwhile, the MAS algorithm demonstrates compression ratios of 11.9%, 11.57%, 12.47%, 12.32% and 12.28% respectively.

In Fig. 4b, EMG data of twelve muscle activities during treadmill walking have been compressed by the MAS and O-MAS-R algorithms. The EMG values are float type that contains 4 bytes per sample. Each EMG dataset comprises 15,000 samples that occupy 60,000 bytes of memory. The RF activity shows the highest O-MAS-R compression ratio of 39.85%, while the MAS is 31.26%. For each group, the O-MAS-R algorithm achieves compression ratios of 39.85%, 35.44%, 34.74%, 39.5%, 35.58%, 36.4%, 33.21%, 36.01%, 39.33%, 35.71%, 35.86% and 37.87% respectively. Meanwhile, the MAS algorithm demonstrates compression ratios of 31.26%, 26.41%, 26.07%, 30.8%, 27.07%, 27.62%, 25.95%, 28.53%, 31.18%, 28.27%, 28.41% and 28.95% respectively.

In Fig. 4c, the compression algorithms have been applied to three accelerometer datasets. The data type in the dataset is float type and contains 4 bytes per sample. Each Accelerometer dataset has 15,000 samples that take 60,000 bytes of memory. For each group, the O-MAS-R algorithm achieves compression ratios of 84%, 83.83%, and 83.76% respectively. Meanwhile, the MAS algorithm demonstrates compression ratios of 38.83%, 38.28%, and 38.77% respectively.

For all the datasets, O-MAS-R compression algorithm demonstrates a better performance. The average increase of O-MAS-R over MAS is shown in Fig. 4d. The accelerometer datasets of O-MAS-R algorithm shows the greatest increase of 45.25% over the MAS algorithm. The average increases of compression ratios for ECG, EMG, and Acc datasets are 7.21%, 8.26%, and 45.25%, respectively.

According to the Spyder platform's profiler tool, the encoding function of the MAS and O-MAS-R algorithms in compressing ECG dataset values took 20.28 µs and 25.69 µs, respectively. However, the repetition of data, on the other hand, resulted in fewer calls to the encoding function in the O-MAS-R algorithm, which decreased the overall run time of the O-MAS-R algorithm. The total run time for the MAS and O-MAS-R algorithms applied in ECG dataset were 79.37 ms and 73.04 ms, respectively. Similarly, the encoding function of the MAS and O-MAS-R algorithms in compressing Accelerometer dataset values took 18.90us and 19.25 µs, respectively. However, due to high frequency of repetitions of data in accelerometer dataset, the total run time for O-MAS-R encoding algorithm is significantly reduced from 283.53 to 71.67 ms^25^.

### MATLAB graphic user interface (GUI)

This paper discusses the smart wheelchair prototype and the three integrated biophysical sensors used to collect four vital health indicators from users. It also discusses the MATLAB GUI software designed to synchronize and download the patients’ healthcare data for diagnosis and analysis.

**Figure 5:**
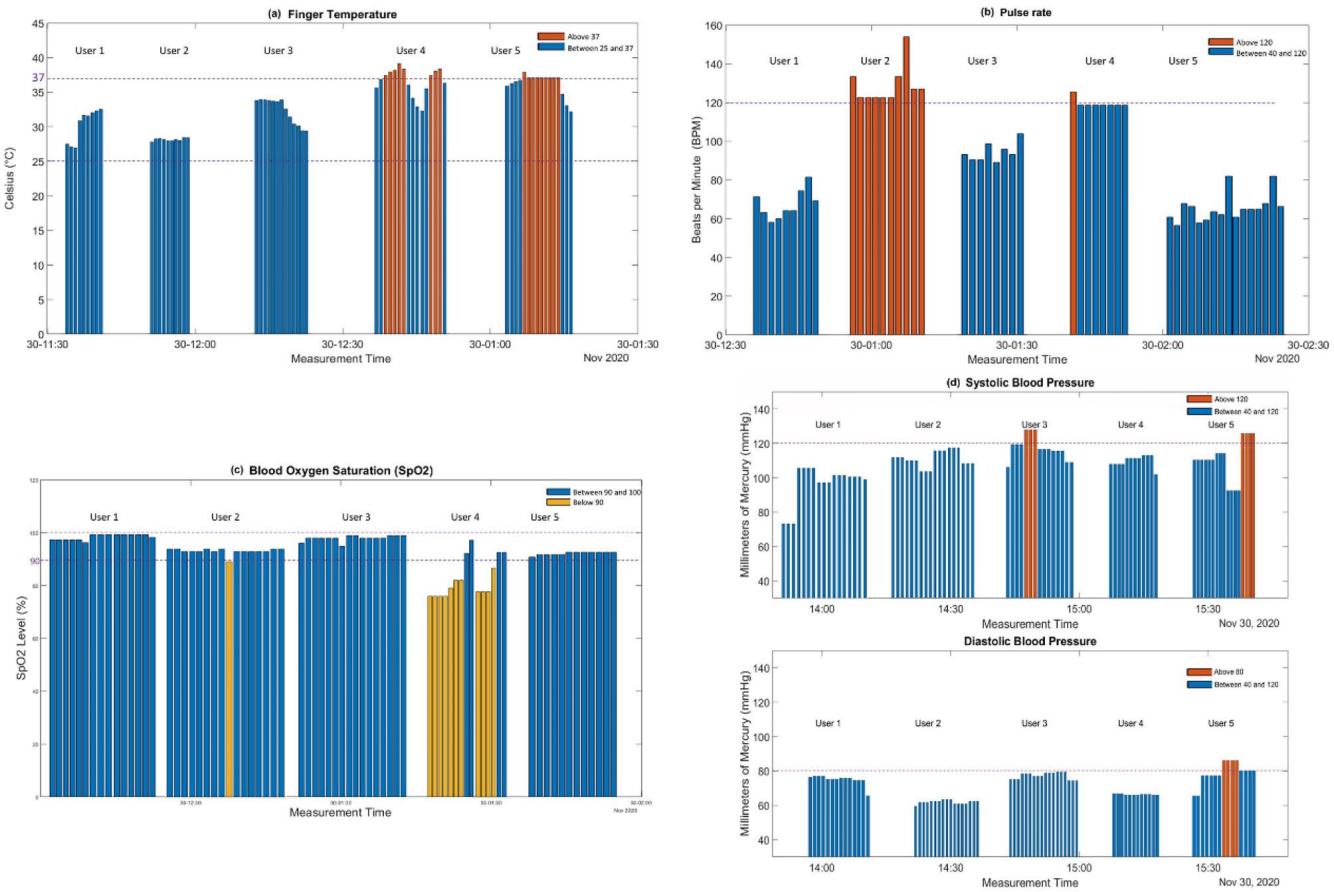
Four types of vital signs from five participants were monitored: (**a**) finger temperature, (**b**) pulse rate, (**c**) blood oxygen levels, and (**d**) blood pressure. Each column represents a single measurement, and the group of columns represents the results from a single participant. The gap between each column is the time spent uploading the measurements.

The preliminary experiments, five participants were involved in the clinical trials, and healthcare data was collected for 5–10 mins for each user. Figure 5a–d demonstrates the results.

Figure 5a documents the five participants whose finger temperatures were measured and recorded. The x-axis is the measurement time, and the y-axis is the measured temperature in Celsius (°C). Before taking the measurements, participants were advised to place their forefinger on their wrist for a minute to equalize the temperature. An upper threshold of 37 °C was set as it was considered as the average normal body temperature. Among the participants, users four and five had a slightly higher temperature than normal, and thus the column automatically turned red following the three-color system.

As seen in Fig. 5b, the five participants’ pulse rate were recorded with the upper threshold set to 120 bpm. The results revealed one participant had a higher average pulse rate than the other participants. Figure 5c depicts the blood oxygen saturation level (SpO_2_) for each participant. The lower limit of SpO_2_ was set at 90%, as any number below that represents hypoxemia, and poses a variety of complications^32^. Therefore, the level of SpO_2_ is a highly useful approach for measuring health conditions^32^. Figure 5d shows the participants’ systolic and diastolic blood pressures in the top and bottom rows, respectively. The upper threshold for systolic blood pressure is 120 mmHg, while the upper threshold for diastolic blood pressure is 80 mmHg. The results indicate that participant three had unreasonably high systolic blood pressure on certain tests, and participant five had high systolic blood pressure and diastolic blood pressure. The three-color system automatically marked the column for high blood pressure data in red.

### iChair autonomous driving

**Figure 6:**
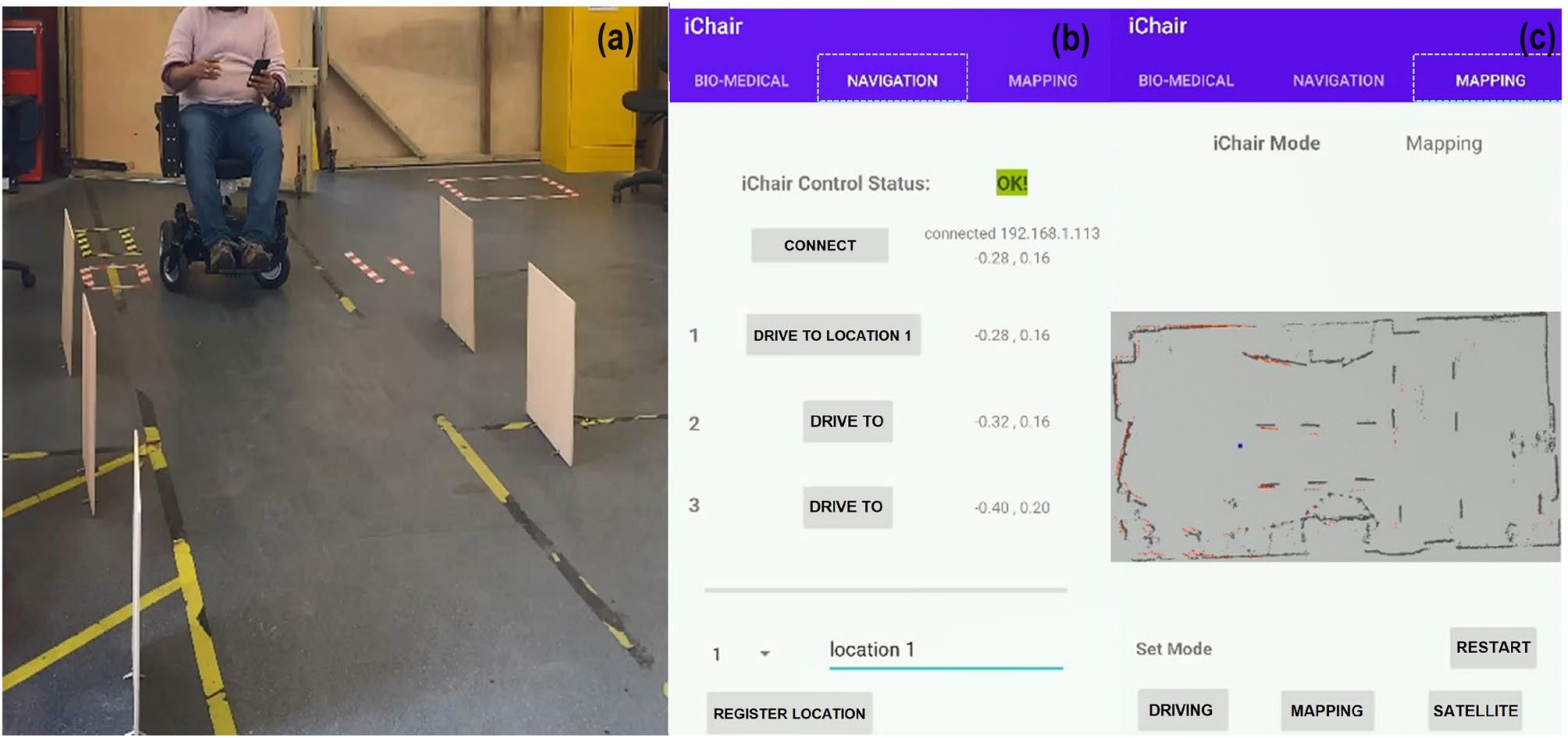
The smart wheelchair autonomous driving and control. (**a**) An engineer sits in the wheelchair and controls it using the iChair app. (**b**) The navigation panel with the iChair app control information, while (**c**) depicts the mapping information of the enclosed area.

The autonomous driving experiments were conducted in the factory testing area^33^. We described the smart wheelchair safety and obstacle detection system in our previously published papers^31^. Based on that system, the wheelchair was improved to travel autonomously from point to point inside a lager and obstacle completed area. An Android-based smartphone app iChair was developed to control and tracks the entire driving progress depicted in Fig. 6.

There are three main sections in the iChair app: bio-medical, navigation and mapping. The biomedical section displays the collected bio-sensory data, the navigation section links the wheelchair to the app and controls its movement, and the mapping section displays the wheelchair's real-time location.

In Fig. 6a, an engineer sits in the wheelchair and controls it using the iChair app. To perform autonomous driving well, the iChair must be in a pre-scanned, enclosed environment, achieved by recording the surrounding information into the map using the data from LIDAR sensors. As shown in Fig. 6c, the app remembers its scanned path of the office, the start and stop coordinates, and the blue dots provides the position of the wheelchair. The red and grey dots, in addition to the lines, are the LIDAR sensors reflecting signals that represent the barriers along the path. Once the scanned map saves, the iChair will link with the app to perform the autonomous driving as shown in Fig. 6b. As a result, the user can enter the start and stop coordinates from the Android app or directly through the ROS network as separate position names. By clicking different positions in the app panel, the wheelchair will drive to the location autonomously.

During the reliability tests, the iChair navigated to various predetermined locations using automated driving scripts. It successfully operated for five hours until the battery ran out of power. Wooden boards were used to modify the configuration of the path during the mobility tests to determine the maximum capacity of the system to maneuver. The results show that the iChair could pass through a minimal gap of 0.85 m and can operate in at least 1.2 m wide corridors. The maximum speed that the wheelchair could move in an unmapped area while accounting for unknown obstacles was 0.2 m/s.

## Discussion

Patients who cannot safely and independently operate an Electric Powered Wheelchair (EPW) must be seated in a manual wheelchair and pushed by a caregiver. An autonomous telemedicine wheelchair is one solution to overcoming the cognitive and physical challenges and improve independence for those users^34^. It not only takes people to their desired location but also assesses their physical location, status conditions and vital bio-signs in real-time. This data will help them manage and prevent chronic diseases in the long term.

The paper proposes a smart wheelchair equipped with three biophysical sensors and a novel Internet of Thing (IoT) compression algorithm that monitors and assesses users' physiological and behavioral data in real-time. The iChair design should prioritize simplicity in control to minimize usage barriers, especially for patients who require assistance. They may initially struggle with or forget to use some of the features. To address the issue, the wheelchair controls should be similar to EPWs on the market, facilitating their usage habits. The central screen can serve as a user-friendly dashboard, displaying the patient's current status, providing prompts for necessary measurements, and offering easy navigation to desired locations. It serves as an interface for users to interact with the iChair smoothly. Due to the wheelchair being integrated with advanced components, algorithms, and sensors, if it is deployed in the market on a large scale, maintenance may require specific technical skills. To mitigate this issue, the system should support remote monitoring and diagnostic tools for spotting issues early. It also provides detailed documents with best practices and maintenance guidelines. Lastly, regular training for maintenance staff can be conducted to ensure they can handle any problems effectively.

The smart wheelchair can further develop as a proprietary medical device for autonomous health monitoring and navigation. For example, it will offer those affected by Parkinson’s disease the ability to proactively manage their chronic condition, and help them avoid fainting, which are considered the most common diagnosis for patients attending emergency departments. It will also help maintain their mobility. The artificial intelligence algorithms incorporated into the wheelchair will analyze sensor data and provide feedback in real-time to the user and clinicians on any potential risks to the patient, such as the experience of a sharp and unexpected drop in blood pressure, causing dizziness and an increased risk of fainting. With the assistant of the smart wheelchiar, the ratio of carers to patients can be increased from 1:2.5 to 1:4 or 1:5 for completely disabled people, allowing the cost of carers to be reduced by up to CNY 15–18 k per year. The wheelchair system is estimated to be priced at CNY 8000 (~ £920), and the retrofitted system is priced at CNY 3000 (~ £345). In the UK market, the cost of the systems will be £2500 and £500, respectively.

During the trials, the system could only process up to three sensors simultaneously, because of the microcontroller’s restrictions in supporting concurrent sensor readings from one group of sensors (analog, UART, Bluetooth) to one interface (TFT, Bluetooth, Wi-Fi)^35^. The constraint may limit the system's coverage of health conditions, especially when managing chronic diseases that involve monitoring multiple health indicators. These problems could be optimized by implementing intelligent algorithms that prioritize and cycle through different sets of sensors over time, ensuring continuous monitoring of key health parameters relevant to chronic conditions. Additionally, adapting the system to support sensor modularity and sensor fusion technologies would enable the integration of more sensors. The detecting sensors integrated into the microcontroller could expand to eighteen different functions, including features such as snore monitoring, temperature readings, glucometer readings, ECGs, EMGs, breath monitoring, SpO_2_, blood pressure, airflow, body position, emergency alarms, and room thermometer, providing a more comprehensive view of the patient's health status.

Health monitoring sensors, such as heart rate, blood pressure and temperature sensors, need to be strategically placed for accurate readings while considering user comfort. Integrating sensors without interfering with wheelchair controls is critical. Thus, for the convenience of our wheelchair design, the temperature sensor was placed on the handrail to detect users’ fingers, palm and wrist temperature. However, we acknowledge that environmental factors influencing temperature in these areas may cause variations in sensor readings. Further improvements involve implementing adaptive calibration algorithms that dynamically adjust temperature readings based on environmental conditions. Additionally, to extend the functionality of the wheelchair, certain sensors can be integrated as conformable and wearable patches on the body and be easily removable modular elements. The integration of multiple sensors, including non-contact sensors on the screen, could be applied to offer a comprehensive approach.

However, the effectiveness of the O-MAS-R compression algorithm may be specific to the types of data used in the study. The performance might vary when applied to different types of datasets beyond the scope of the initial experiments. Additionally, the study demonstrated positive results under controlled conditions, but real-world scenarios can be more complex. Factors such as signal interference, hardware malfunctions, or variations in environmental conditions could affect the actual performance of the proposed model.

Further research can focus on optimizing the compression algorithm for diverse sensor data types, ensuring it maintains efficiency across a wide range of physiological parameters. Extensive validation studies can be conducted in diverse healthcare settings, such as different patient demographics, environmental conditions, and healthcare practices. Moreover, the algorithm can be further integrated with advanced healthcare AI models for automated monitoring and forecasting of users' physiological conditions and diseases.

To explore the EPW with other sensors for more functionalities, previous work by Shen et.al.,^36^ extend  the scope of the work. This extension includes a face-recognition screen with a camera on the left handrail of the wheelchair. This innovative approach aims to evaluate users' long-term cardiovascular conditions based on facial information, utilizing a CHD evaluation algorithm published by Shen^36^. First, sixty-eight face feature points and ears from patients’ face images were collected. Based on their coordinates, six regions of interests (ROI) were extracted: left canthus, right canthus, left crowsfeet, right crowsfeet, nose bridge and forehead^36^. Then, a gray-level co-occurrence matrix algorithm was applied to the ROIs to extract and analyze their texture features. Lastly, the random forest and decision tree classification methods were applied to predict the risk of CHD.

In the paper, 1528 facial images were captured from 309 subjects, comprising 226 males and 83 females^36^. Among them, 195 patients have coronary heart disease. Each patient had at least three face images collected: front, left, and right faces. By adopting features into the models, the random forest algorithm had a maximum accuracy of 72.73% in identifying patients with CHD, while the decision tree model had a maximum accuracy of 70.45%. The results demonstrated that facial images can be an effective method of detecting patients with CHD, with an accuracy rate of above 70%. The algorithm will be embedded into the wheelchair's screen to monitor the user’s coronary health condition over time.

In the paper, we demonstrated that the proposed use of the O-MAS-R compression algorithm maintained a greater compression ratio than the MAS algorithm at a 53% reduction in data transmission power consumption^24^. As the compression ratio is directly proportional to data transmission power usage, implementing the O-MAS-R algorithm in wireless sensor network sensor nodes will result in even lower data transmission power consumption^25^. This approach uses the least amount of memory to store and transmit data by reducing consecutively repeated data values. This functionality is particularly useful in dealing with healthcare data. However, the effectiveness of the O-MAS-R compression algorithm may be specific to the types of data used in the study. The performance might vary when applied to different types of datasets beyond the scope of the initial experiments. Additionally, the study demonstrated positive results under controlled conditions, but real-world scenarios can be more complex. Factors such as signal interference, hardware malfunctions, or variations in environmental conditions could affect the actual performance of the proposed model.

This paper documents and evaluates the obstacle avoidance, human–machine interaction, and point-to-point autonomous driving of the smart wheelchair. Currently, the intelligent wheelchair can only drive autonomously in a pre-scanned enclosed area because the only way to calculate the optimal route between any two locations requires the system to store localized data from the laser sensors. However, once scanned, the stored maps and routes can be shared with other wheelchairs for collaborative driving.

For wheelchair users with limited mobility, safety is the top priority. Unmapped areas may have construction zones, temporary obstacles, changes in road conditions, lacking lane markings and road signs, which can cause severe dangers to the wheelchair's autonomous driving. Therefore, the autonomous driving function will be deactivated in unmapped areas. Users have to rely on the manual control of the wheelchair to ensure safety. Additionally, to ensure safety for wheelchair users, we conduct thorough testing to validate the system's performance under different conditions, ensuring robustness and safety. We implement redundant sensor systems, the obstacle avoidance system, to ensure the vehicle can rely on multiple sources of information, mitigating the risk of sensor failures and avoiding collisions. A software filter that used LIDAR sensor data successfully hid the user’s legs from the scan data to minimize blind spots. Increasing the use of obstacle detection over a wider range reduced the remaining blind spots discovered around the four corners of the wheelchair.

The smart autonomous wheelchair will assist disabled and elderly patients by allowing them to pick locations on their phones and drive independently and autonomously. It will reduce their dependency on caregivers and family members while also eliciting feelings of self-reliance. Therefore, the wheelchair has potential uses in nursing homes, hospitals, communities, airports, and shopping malls. In hospitals and nursing homes, the wheelchair will work in conjunction with the other infrastructure, such as elevators, ward doors, and automated doors to complete easy point-to-point and ward-to-ward mobility. The telemedicine diagnosis from the wheelchair will complete the initial evaluation of vital sign measurements at the hospital’s entrance and then continually monitor those patients.

## Conclusion

In this paper we proposed a smart autonomous wheelchair (iChair) that integrates with telemedicine sensors based on IoT. The wheelchair, controlled by a mobile app, achieved point-to-point autonomous driving within a predefined area with and without obstructions. Various sensors, including wireless location, position accelerometer, seat cushion sensors, and biophysical sensors embedded in the wheelchair, collected users’ physiological and behavioral data in real-time. This comprehensive data was extracted, transformed, and uploaded to a cloud platform for storage. An improved data compression algorithm, Minimalist, Adaptive and Streaming R-bit (O-MAS-R) will likely achieve a higher compression ratio during the data transmission. Performance of MAS and O-MAS-R was evaluated in healthcare applications such as ECG, EMG, and accelerometer datasets. The designed user interface allowed users and their caretakers or doctors to see and analyze the data using the artificial intelligence algorithm to receive telediagnosis, advice and alerts. The interface also allowed users to track and diagnose long-term health issues with similar algorithms and makes it easier for medical professionals to diagnose probable health conditions in the patients.

## Methods

### System architecture

The robotic wheelchair system was designed based on the research of our previously published papers^31^.

The wheelchair prototype modified and improved upon the Titan-LTE powered wheelchair^37^ and integrated with the DMC60C digital motor controllers^38^ to allow wheelchair manipulation both manually and autonomously. The new components include DC motor controllers, a Jetson Nano developer kit, an Inertial Measurement Unit (IMU), a joystick module, two light detection and ranging sensors (LIDAR), and a 3D printed shield were incorporated into the wheelchair and allowed users to operate the wheelchair via a mobile app. These integrated components communicated with each other by a central Controller Area Network (CAN). The joystick module was a custom-made unit that used a potentiometer joystick with access to the CAN enabled microcontroller.

The Jetson module included Wi-Fi capability, which allowed the entire wheelchair system to be linked to a wireless Android application. The software that enabled mobility assistance and autonomous driving was written in C++. The sensors connected to the Jetson Nano development kit used Robot Operating System middleware (ROS). It implemented a navigation stack and custom configurations for obstacle avoidance. The stack consisted of specially developed modules, including a localization module and a mapping module. The packages for reading the joystick, movement aid, and motor control were developed while the autonomous movement was powered by an open-source navigation. The Timed Elastic-Band (TEB) route planner^30^ enabled path planning optimization to ensure smooth and safe mobility in the iChair system. It also included two laser sensors^31^ mounted on the front of the wheelchair to help ensure obstruct avoidance.

The microcontroller used by the data acquisition unit was an Arduino component^39^, while the biophysical sensors were MySignals packages^35^. Consequently, we designed a converter microcontroller to resolve the incompatibility between the Arduino and MySignals system. The ThingSpeak^40^ cloud platform was used to allow users to view, download and analyze the stored data. We also developed a new MATLAB graphic user interface (GUI) to help users and doctors access and diagnose health information in real-time.

### Data communication and compression

We introduced the proposed Minimalist, Adaptive and Streaming R-bit (O-MAS-R) data compression algorithm in our previously published papers^25,31^. The improvements made to the MAS algorithm allowed for a decrease in the sequential repeating of data values, which lead to a higher compression ratio. Equation (1) represents the floating data format of the O-MAS-R data compression algorithm.

1 $${\text{nnn}}/{\text{eee}}/{\text{ns}}/ \ldots {\text{input data}} \ldots /{\text{R}}/ \ldots$$

where nnn is the length of the input data in binary format, eee represents the position of the decimal point for the input data from left to right. Additionally, ns shows whether the input value is positive or negative, and the proposed R-bit represents the consecutive repetition of input digits.

The algorithm calculates up to seven input digits. The repetition input value from the subsequent input data sets the R digit to 1. When there is no repetition, R is 0. The number of R-bits increases as the number of consecutive repetitions of input data increases. The decoding process outputs the same value until it reads 0. Similarly, Eq. (2) represents the O-MAS-R encoding format for the integer value.

2 $$000/{\text{nnn}}/{\text{ns}}/ \ldots {\text{input data}} \ldots /{\text{R}}/ \ldots$$

To distinguish between integer and floating-point data, the first three digits 000 indicated the input data is integer and eee bit is removed. The repetition digit R-bit indicates if the following data is the same as the current value.

The following describes the detailed encoding and decoding process of data. When the sensor nodes send out data, the algorithm determines if the value is an integer or float number. When the value is a float number, the data value compresses using the float encoding format described in Eq. (1). In contrast, when the value is an integer, the integer encoding format [Eq. (2)] compresses the data. Following data encoding, an R-bit will append to the end of the format dependent on the repetition of the next data value. If the value is the same as the present value, the R-bit is 1. If not, the R-bit value is 0. For the data decoding progress, the software reads the first data value and examines the R-bit to determine whether the upcoming value is the same as the current value. If the R-bit is 1, the upcoming value is treated as the same as the current one. The method keeps reading R-bit until it equals 0.

Both the MAS and O-MAS-R were implemented across three healthcare datasets: electrocardiography (ECG), surface electromyography (sEMG), and accelerometer-based events (Acc) to assess the efficacy of the data compression methods. Scripts for data compression algorithms were simulated in Spyder (Python 3.7). The compression ratio was determined by dividing the dataset’s compressed size by its original size, as indicated in Eq. (3). The higher the compression ratio, the better the data compression algorithm would perform.

3 $${\text{Compression}}\;{\text{ratio }} = \, \left( {{\text{Compressed}}\;{\text{size}}} \right) \, / \, \left( {{\text{Original}}\;{\text{size}}} \right) \, \times { 1}00\%$$

Five ECG datasets^41^, twelve EMG datasets^42^, and three accelerometer datasets^43^ were obtained from the MIT-BIH Arrhythmia Database^44^, with a sampling frequency of 360 samples per second and an 11-bit resolution. Additionally, sEMG datasets were recorded at 1.5 kHz, corresponding to 12 lower limb muscles in a healthy subject during treadmill walking. These muscles include rectus gemoris (RF), vastus lateralis (VL), gracilis (GR), biceps femoris long head (BFLH), tensor fasciae latae (TFL), Vastus medialis (VM), Tibialis Anterior (TA), Soleus (SOL), Gluteus Medius (GMD), gastrocnemius lateralis (GL), gastrocnemius medialis (GM) and semitendinosus (SEM)^44^. Lastly, datasets from three-axis accelerometers were selected and evaluated at a frequency of 120 Hz.

### Ethical approval

This study was approved by the Innovative Technology and Science Ltd on 2020.06. We affirm that all experiments were conducted in compliance with the experimental guidelines and regulations established by the Innovative Technology and Science Ltd.

## Data Availability

The data that support the findings of this study are available from the corresponding author, upon reasonable request.
